# Using word-picture verification to inform language impairment locus in chronic post-stroke aphasia

**DOI:** 10.3389/fresc.2022.1012588

**Published:** 2022-10-31

**Authors:** Alexandra Z. Durfee, Stacy M. Harnish

**Affiliations:** ^1^ Stroke Cognitive Outcomes and Recovery Laboratory, Department of Neurology, Johns Hopkins University School of Medicine, Baltimore, MD, United States; ^2^ Aphasia Laboratory, Department of Speech and Hearing Science, The Ohio State University, Columbus, OH, United States

**Keywords:** stroke, aphasia, word-picture verification, semantics, phonology, impairment locus

## Abstract

Word-picture verification, a task that requires a yes/no response to whether a word and a picture match, has been used for both receptive and expressive language; however, there is limited systematic investigation on the linguistic subprocesses targeted by the task. Verification may help to identify linguistic strengths and weaknesses to ultimately provide more targeted, individualized lexical retrieval intervention. The current study assessed the association of semantic and phonological skills with verification performance to demonstrate early efficacy of the paradigm as an aphasia assessment. Sixteen adults with chronic post-stroke aphasia completed a battery of language assessments in addition to reading and auditory verification tasks. Verification scores were positively correlated with auditory and reading comprehension. Accuracy of semantic and phonological verification were positively correlated with accuracy on respective receptive language tasks. More semantic errors were made during verification than naming. The relationship of phonological errors between naming and verification varied by modality (reading or listening). Semantic and phonological performance significantly predicted verification response accuracy and latency. In sum, we propose that verification tasks are particularly useful because they inform semantics pre-lemma selection and phonological decoding, helping to localize individual linguistic strengths and weaknesses, especially in the presence of significant motor speech impairment that can obscure expressive language abilities.

## 1 Introduction

**Figure 1:**
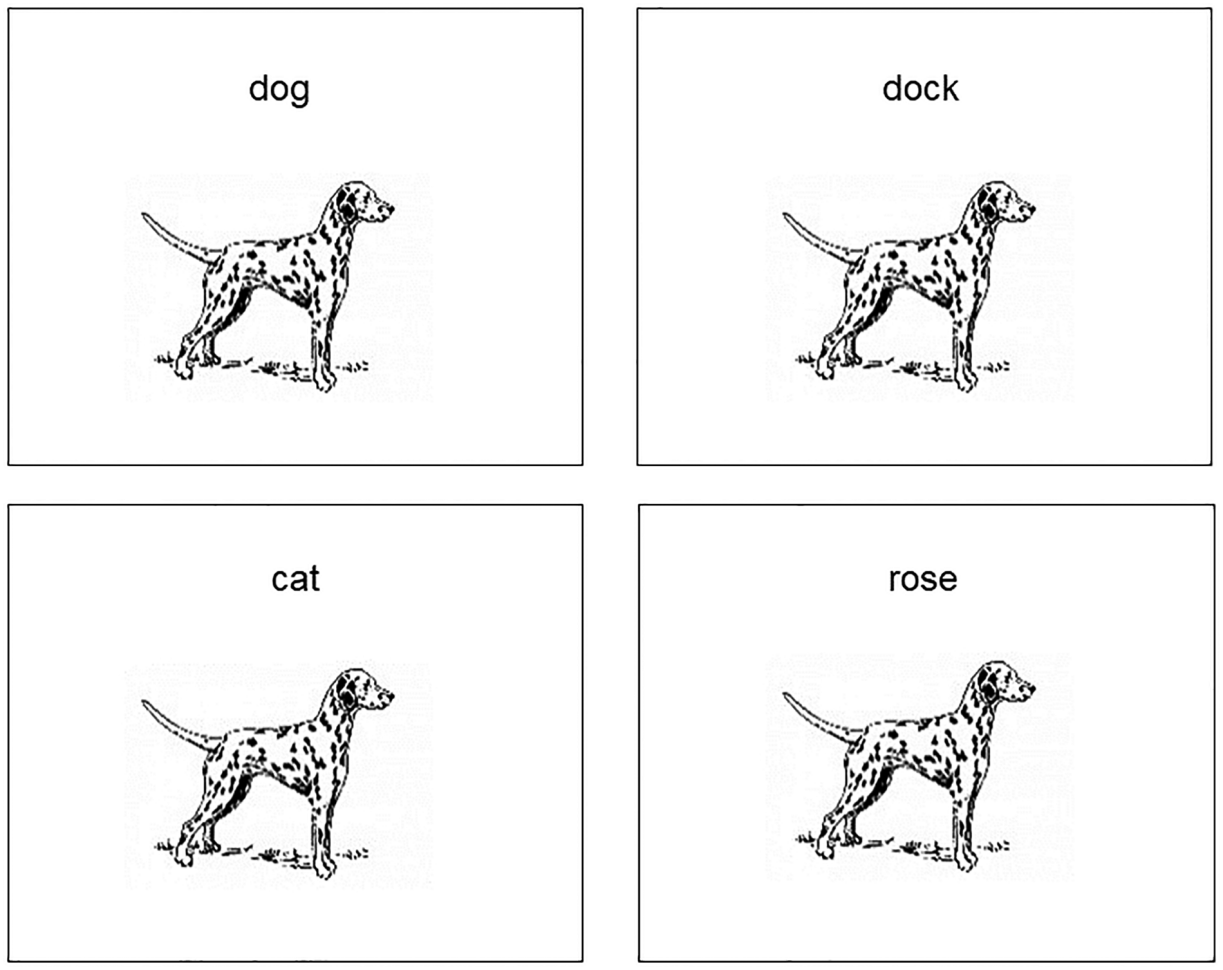
Example of separate rWPVT trials for a single picture with the target (top left), phonological foil (top right), semantic foil (bottom left), and unrelated foil (bottom right).

Word-picture verification tasks (WPVTs) are a versatile language tool used with both healthy adults (1–3) and individuals with aphasia (IWAs) (4–7). Verification, wherein a picture is presented with a word and an individual makes a yes/no judgment if the presented word-picture pair matches or does not match (see Figure 1), is more sensitive to comprehension deficits compared to multiple choice (4). When a single picture is presented with a four-word array, such as in typical word-picture matching tasks, the likelihood of selecting the correct answer by chance is 25%. When that same picture and set of words is presented in the verification paradigm, the likelihood of correct referent selection by chance drops to 6.25%. WPVT performance differentiated healthy adults and IWAs, with IWAs consistently responding less accurately on trials including phonological and semantic competitors (8). Thus, the paradigm appears to provide insight into semantic (9) and phonological abilities at the single word level. Therapeutically, verification has been used to target both receptive (6, 7) and expressive (10–12) language processing at the single word level. Such a task that seemingly targest multiple language domains (i.e., expressive and receptive language) and subprocesses of language (i.e., phonology, semantics) would prove advantageous to maximize efficiency for language assessment and treatment.

Despite available evidence in the literature on WPVT performance in healthy adults and IWAs, there is still a dearth of knowledge about the paradigm’s theoretical underpinnings to inform its use as a model-driven, empirically-based assessment tool. Previous work with healthy adults concluded that WPVT performance provides information on language production-based, pre-lexical conceptuo/perceptuo-semantic abilities (2). Still, other work with IWAs has indicated that the task taps into comprehension-based lexical-semantic processes (4) but that performance is also related to production-based performance (9). Based on Indefrey and Levelt’s models of language processing (13), conceptual and lemma [i.e., the form of a word that contains the conceptual-semantic, and morphological coding prior to phonological encoding (14)] information are shared between language comprehension and production. The Organised Unitary Content Hypothesis (OUCH (9, 15) also suggests that semantic information is stored in an amodal hub, and that language production and comprehension processes access the hub (i.e., semantic information). Semantic processing has received more attention compared to phonological processing as it relates to verification performance (9, 16). *WPVT performance and its relationship with language comprehension and production as well as phonological processing skills have yet to be investigated together in one study*.

Many WPVT studies utilized a varying combination of word-picture pairs, rendering cross-study comparison a challenge. Some studies have included two contexts – matched word-picture pairs and unrelated unmatched word-picture pairs (2, 17). Still, other studies include linguistically-related unmatched pairs along with matched pairs (1, 4, 5, 9, 16, 18). Including matched and unmatched conditions that convey a variety of linguistic relationships could provide a more holistic picture of intact and disrupted language skills by requiring “finer” linguistic processing compared to verification versions that utilize target and unrelated word-picture pair relationships (and thus demand a coarser processing of linguistic information). For instance, an individual who consistently, and accurately, indicates that an unrelated (e.g., *girl-saw*) and semantically-related (e.g., *hammer-saw*) word do not match the presented picture but is incorrect to do so with the phonologically-related (e.g., *salt-saw*) word may indicate a relative deficit with phonology that would not have otherwise been as evident without the varying linguistic contexts.

Previous WPVT work has focused on accuracy performance only. In certain scenarios, it may be the case that accuracy alone does not paint the most complete picture of an individual’s language processing abilities or disabilities. For instance, if a healthy aging adult was shown a picture of a cat, it is likely that they would be able to produce the target picture name in under five seconds. However, perhaps when an IWA is presented with a picture of a cat, it takes 30 s for him/her to correctly produce the target. If only accuracy were collected, both individuals would appear to have identical performance. However, if response latency was taken into consideration, the IWA’s response would indicate an impairment compared to the healthy aging adult. Thus, incorporation of reaction time data, which has had limited attention in the WPVT literature among IWAs, may provide further insight into language processing abilities (19), especially among those individuals who score above the cutoff for impairment on traditional aphasia tests but who report continued language difficulties.

In clinical and research speech-language pathology, there is a need for continued development of model-driven language assessments. Assessments that reduce or eliminate the potential confound of motor speech impairment, which commonly co-occurs with language deficits (20), are also vital to identify speech versus language impairment locus. That is, this WPVT could assist with differential diagnosis of motor speech and aphasia when used in tandem with other speech and language measures. In settings where administration of standardized assessments is not possible due to time constraints, purchased testing material availability, etc., a theoretically-driven assessment that taps into receptive and expressive language along with language subprocesses (e.g., semantics, phonology, lexicon) would be both efficient and efficacious, as limited aphasia assessments are available that are both brief and developed with consideration for empirically-supported models of language processing. Additionally, some of these assessments have been criticized for their weak psychometric properties or lack of theoretical basis of language processing. For instance, the Aphasia Quotient of the Western Aphasia Battery (21) is more heavily influenced by performance on production over comprehension sections (22).

The current study aims to determine the relationship between WPVT performance and performance on established measures of expressive and receptive language in addition to tasks that more finely target semantic and phonological processing among adults with chronic post-stroke aphasia. We hypothesize that WPVT performance will be associated with performance on established measures of overall receptive language ability as well as on established receptive language tasks focusing on semantic and phonological processing. Since previous work observed no difference in semantic errors during verification and naming in a single case study (9), we hypothesize that semantic errors will not differ between verification and naming in a larger group of individuals with aphasia. Additionally, we hypothesize that phonological errors between naming and verification will also not differ. Finally, we hypothesize that performance on established measures of semantic and phonological processing will predict accuracy and response latency on WPVTs. Findings will replicate previous findings of WPVT and confrontation naming relationships observed in a single case study (9) and contribute to the corpus of language assessments in development for research and clinical speech-language pathology use. If a relationship is observed between WPVT performance and performance on established measures of language comprehension and production, then the WPVT could be used as a tool to identify locus of language impairment in IWAs while circumventing the motor speech system. Such information would prove vital in research settings, such as to determine profiles of responders and non-responders to an experimental aphasia treatment (23), and in clinical settings, such as to inform treatment planning for aphasia rehabilitation.

## 2 Materials and methods

### 2.1 Participants

The study was approved by The Ohio State University Institutional Review Board prior to participant recruitment. Participants provided informed written consent prior to participation in study activities. IWAs had a left cerebral hemisphere stroke at least six months prior as indicated by interview and medical record review. They did not have suspected diffuse brain injury or disease as indicated *via* medical record review and self- or caregiver-support report. All IWAs scored within the impairment range (i.e., mean modality T score < 62.8) on the *Comprehensive Aphasia Test (CAT)* (24). Via self- or caregiver-supported report, IWAs indicated that English was the primary language learned while developing language as a child. IWAs also reported that they were right-handed prior to their stroke as indicated by reporting use of their right hand on at least 6/10 scenarios (e.g., using a toothbrush) on a modified version of the *Edinburgh Handedness Inventory* (25).

Data from participants who reported illegal substance use or substance abuse in the past month was compared to the rest of the data set to check for outliers in performance, as there is a paucity of research regarding its impact on strictly language performance, and what evidence exists is mixed and complex regarding its impact on cognitive-linguistic skills (26–29). Substance abuse included (1) heavy alcohol use, which was defined as “five or more drinks for males and four or more drinks for females within a few hours on five or more days in the past month” [“binge drinking” (30)], and (2) substance abuse, which was defined as use of any illegal drug or a legal drug in a way not prescribed by a physician. We observed no outlier in performance among those who reported illegal substance use or substance abuse compared to those who reported a recent negative history, so data from participants who reported a positive drug history were included in the final dataset.

Hearing, vision/neglect, and depression were screened prior to completion of experimental speech-language tasks. Hearing acuity was screened *via* pure tone audiometry at 500, 1000, and 2000 Hz. For participants younger than 50 years of age, all frequencies were presented at 20 dB HL. For participants 50 years of age or older, 500 Hz tones were presented at 20 dB HL, and 1,000 and 2000 Hz were presented at 40 dB HL to account for possible age-related hearing changes. IWAs without hearing aids were assessed with over-the-ear headphones, and IWAs with hearing aids were tested within the sound field. Tones were presented to the right and left ear separately when headphones were used. IWAs must have indicated that they heard all tones presented to both ears to be included in study analyses.

Vision acuity was assessed *via* the *Lea Symbols Line Test* (31). A visual aid including enlarged pictures of the symbols on the card along with the corresponding name printed below the symbol was utilized to minimize the impact of speech-language impairment on vision screening performance. To be included in study analyses, IWAs accurately identified the symbols on the 20/100 line of the card, presented at approximately 16 inches. Visual neglect was also screened using a modified version of the *Alberts Test* (32), which required individuals to cross out a series of lines presented on a page. To be included in study analyses, IWAs must not have missed crossing out more than one line on the page.

Depression was screened using the *Patient Health Questionnaire-8 (PHQ-8)*. IWAs indicated that they did not have severe depression (*PHQ-8* score < 20) to have their data included in study analyses.

### 2.2 Stimuli and procedures

All behavioral testing was completed in one session up to three hours in duration. Breaks were provided throughout testing sessions at participants’ request or the examiner’s discretion.

#### 2.2.1 Motor speech

IWAs’ motor speech abilities were judged based on presence and severity *via* completion of a series of verbal tasks. Some tasks were adapted from the *Apraxia Battery for Adults* (33) and included a measure of diadochokinetic rate, repetition of words of increasing length (e.g., thick, thicken, thickening), automatic speech tasks (e.g., listing the days of the week), repetition of multisyllabic words (e.g., saying motorcycle three times in a row), and single and multisyllabic reading of words. Two certified speech-language pathologists blinded to participant identity listened to audio recordings of the motor speech tasks and rated the presence and severity of dysarthria and apraxia of speech. Ratings of dysarthria and apraxia of speech severity were completed on separate scales, which ranged on a scale from zero (absence of motor speech impairment) to seven (profound). Overall motor speech ability was calculated by taking the median of dysarthria and apraxia of speech scores across raters.

#### 2.2.2 Aphasia severity

The *CAT* was employed as a measure of presence and severity of aphasia. All eight language subtests from the *CAT* were administered to participants and included (1) comprehension of spoken language, (2) comprehension of written language, (3) repetition, (4) spoken naming, (5) spoken picture description, (6) reading aloud, (7) writing, and (8) written picture description. Each subtest yielded a *T* score, or standard score, and taking the mean of the eight subtest *T* scores produced the mean modality *T* score. According to the *CAT*’s manual, a mean modality *T* score cutoff of 62.8 (i.e., scores that fell below 62.8) correctly identified about 91% of IWAs as having aphasia and thus was used as the cutoff score to indicate aphasia in the current study (24).

#### 2.2.3 Receptive language

*T* scores from the comprehension of spoken language and comprehension of written language subtests from the *CAT* were used as measures of IWAs’ comprehension abilities. Comprehension of spoken language *T* scores were derived tasks of comprehension at the word, sentence, and paragraph (discourse) level, and comprehension of written language *T* scores were derived from tasks targeting comprehension at the word and sentence level.

##### 2.2.3.1 Receptive phonology

**Figure 2:**
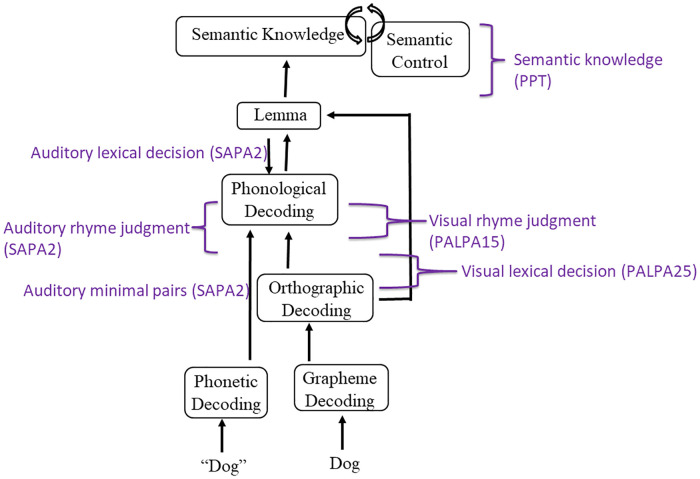
Model of receptive language and associated assessments. Model is informed by language processing models by Dell and colleagues (14), Foygel and Dell (34), and Indefrey and Levelt (13).

Three tasks investigating receptive phonological processing were completed by IWAs based on the levels of phonological decoding included in our model (See Figure 2). According to Martin and Saffran (35), minimal pair processing reflects the mapping of acoustic/phonetic information onto phonemic/phonological codes. Auditory rhyme judgment targets acoustic-to-phoneme mapping at a later decoding stage compared to minimal pairs and requires manipulation of the phonological form. Auditory lexical decision is believed to assess the mapping of phonological to lexical representations. Subtest 2 from the *Standardized Assessment of Phonology in Aphasia* (*SAPA2*) (36) was administered to provide a measure of auditory-based phonological processing skills. Four sections comprises *SAPA2*, assessing real word and non-word rhyme judgment, lexical decision, and minimal pair judgment.

Visual rhyme judgment requires access to phonological input codes from orthography (37, 38) and perhaps expressive phonological codes as well as expressive-receptive language connections (37). Visual lexical decision evaluates the integrity of orthographic representations (39–41) and their connections to phonological representations (39, 41, 42).^1^ Semantic processing may also be tapped into, such as in the presence of pseudohomophones with similar orthographic characteristics (e.g., *soap – sope*) (42). Taken together, both auditory and reading phonological tasks appear to target processes of interest for WPVT performance, specifically phonological representations and their connections to lexicons. *Psycholinguistic Assessment of Language Processing in Aphasia* (*PALPA*) (43) subtests #15 (written word rhyme judgments) and #25 (visual lexical decision) were administered to investigate IWAs’ input phonological processing from orthography. The included *PALPA* subtests were chosen as they closely mirrored three of the four sections in *SAPA2*.

Phonological processing scores were converted to proportions using a-prime calculations (44), as this measure has been argued to be more sensitive to hit (i.e., correct endorsement of the target) and false alarm (i.e., incorrect endorsement of a non-target) ratios in relation to response bias as discussed in Signal Detection Theory (44). A-prime was selected over d-prime as it is more easily interpretable (16, 44, 45). A-prime values range from 0.50-1.00, with values closer to 0.50 indicative of chance performance, or performance that is undistinguishable from noise. A-prime scores were calculated for receptive auditory (i.e., *SAPA2*) and reading (i.e., *PALPA*) performance. An average was taken of each *PALPA* subtest a-prime score to generate a single score.

##### 2.2.3.2 Receptive semantics

The three-picture version of the *Pyramids and Palm Trees test* (*PPT*) (46) was utilized as a measure of conceptual semantic processing. In this *PPT* version, one black-and-white picture is presented at the top of the page, and two black-and-white pictures are presented at the bottom of the page. The two pictures at the bottom of the page are semantic coordinates (i.e., from the same semantic category) whereas the top picture is typically from a separate category. IWAs were asked to choose which of the two pictures at the bottom of the page shared a semantic relationship (e.g., property, association) with the top picture. According to the *PPT* manual, performance on the picture version of the *PPT* assesses object recognition, access of object semantic information, and object semantic system integrity (46).

Figure 2 displays the receptive language tasks employed and the level of language processing they were intended to target.

#### 2.2.4 Expressive language (lexical retrieval)

The *Boston Naming Test* (*BNT*) (47) was employed as a measure of lexical retrieval in IWAs. *BNT* stimuli consist of black-and-white line drawings of objects, ranging from high to low frequency. IWAs were presented with a picture and asked to produce the picture name to the best of their ability. Responses were scored as correct if IWAs accurately verbalized the name of the picture independently or following a semantic cue. Phonemic distortions or the addition of –*s* at the end of picture names that did not change the meaning of the name were scored as correct. Responses were scored as incorrect if IWAs were unable to produce the picture name independently or were able to accurately name the picture following a phonemic cue, in accordance with *BNT* scoring procedures. However, *BNT* administration differed in the current study from the assessment manual in that all trials were administered to participants and were not discontinued for observed floor effects.

##### 2.2.4.1 Expressive phonology and semantics

Verbal responses on the *BNT* were analyzed for phonological- and semantic-based errors. Errors were considered phonological in nature if IWAs’ responses contained a phonemic substitution (e.g., *tat* for *cat*), omission (e.g., *at* for *cat*), addition (e.g., *scat* for *cat*), or transposition (e.g., *tac/tack* for *cat*) as described in scoring procedures provided online for the *Philadelphia Naming Test* (48) (phonological error classification sheet taken from https://mrri.org/philadelphia-naming-test/). Errors were deemed semantic in nature if they appeared to share a coordinate (e.g., *dog* for *cat*), superordinate (e.g., *pet* for *cat*), subordinate (e.g., *tabby* for *cat*), or associative (e.g., *whiskers* for *cat*) relationship with the picture name. Errors that appeared to share features of both phonological- and semantic-based errors were considered mixed errors.

Semantic, phonological, and mixed errors along with non-word and unrelated errors were used in the calculation of s-weight and *p*-weight, which are measures of the connection weights between the semantic and lexical level as well as the lexical and phonological level, respectively. These weight connections are based on Dell’s interactive activation model of lexical retrieval (14), wherein a picture name is ultimately selected depending on the degree of automatic spreading activation between the conceptual, lexical, and phonological language production stages. A low s-weight is indicative of a semantic-based impairment, or an impairment linking the semantic and lexical levels, resulting in more semantic-based errors in naming. Likewise, a smaller *p*-weight is indicative of a more phonological-based impairment, with more non-word (and phonological paraphasias) observed compared to semantic paraphasias during naming (34, 49). Calculation of s- and *p*-weights was completed with the assistance of an online calculator (http://www.cogsci.uci.edu/∼alns/webfit).

**Figure 3:**
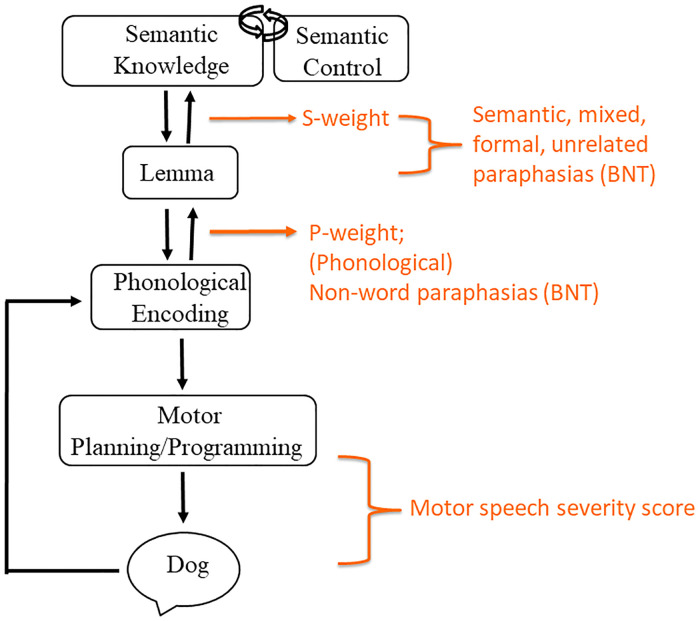
Model of expressive language and associated assessments. Model is informed by language processing models by Dell and colleagues (14), Foygel and Dell (34), and Indefrey and Levelt (13).

Figure 3 displays the expressive language tasks employed and the level of language processing they were intended to target.

#### 2.2.5 WPVT

Pictures and their associated names for the WPVT were taken from the *BNT* after appropriate written authorization was granted by PRO-ED, Inc. for use. On the WPVT, pictures were presented in the middle of the laptop computer screen, with picture size held constant. Each picture was presented with a single word that was either congruent or incongruent (i.e., a word foil) with the picture’s name. These word foils were chosen from the SUBTLEXus frequency database (50) by the first author and discussed with the second author to achieve a consensus on the words’ inclusion as stimuli. Foils consisted of semantically related, phonologically related, and unrelated words to the pictures. Semantic foils shared a coordinate semantic relation with the picture (e.g., a picture of a couch and the word chair). Phonological foils shared at least the first two phonemes with the picture name and were not semantically related to the picture (e.g., a picture of a candle and the word candy). Unrelated foils were not semantically related to the picture (e.g., a picture of a broom and the word fox). Semantic and Unrelated foils also shared less than a 33% phonological overlap with the picture name to reduce the influence of phonological similarity. The foils and the picture name did not significantly differ in terms of lexical frequency [*F*(3,236) = 1.28, *p *= 0.28] (50), imageability [*F*(3,236) = 1.0, *p *= 0.40] (51), or phonotactic probability [*F*(3,236) = 1.38, *p *= 0.25] (52) as well as in the number of letters [*F*(3,236) = 0.06, *p *= 0.98], phonemes [*F*(3,236) = 0.34, *p *= 0.80], and syllables [*F*(3,236) = 0.50, *p *= 0.69].

The WPVT was presented as two versions: auditory (aWPVT) and reading (rWPVT). On the aWPVT, the picture appeared in the middle of the laptop computer screen, and the word was presented 25 ms later. In real time, the onset asynchrony between the picture and the audio recording of the word allowed both stimuli to appear to be presented simultaneously. The picture remained on the screen until participants provided their response *via* button press. Linguistic stimuli were presented only once. Recordings of the words were spoken by a singular female speaker and presented at 70 dB SPL. On the rWPVT, a word appeared in all lower case directly above the picture in size 36 Arial font. Both the word and the picture remained on the screen until participants provided their response *via* button press. No time restraint was placed on linguistic stimuli presentation for the rWPVT to best approximate reading in everyday situations as well as in reading assessments commonly used with IWAs. On both versions of the WPVT, a white blank screen appeared after participants provided their response with an inter-stimulus interval of 2000 ms before presentation of the next trial.

Each picture was presented with its name and its three foils, each on separate trials and randomized with other trials within the presentation format. That is, the foils were not each presented consecutively, rather, they were interspersed with foils and targets from other trials (e.g., [picture of a dog] – CAT, [picture of a table] – TABLE, [picture of a banana] – BANDANA, etc.). Word-picture pair presentation order was randomized. Thus, participants saw each picture (sixty total) four times (one congruent word-picture pair, three incongruent word-picture pairs) in two different presentation formats (auditory and reading) for a total of 480 items. aWPVT and rWPVT presentation order was counterbalanced across participants.

On the aWPVT, participants were told that they would hear a word and see a picture at the same time. They were instructed to respond yes when the picture and word match or no when the picture and word did not match. Responses were made *via* button press using the left (yes responses) and right (no responses) arrows on the laptop keyboard. Visual cues of a green check (yes responses) and red ‘X’ (no responses) were placed below the arrow keys and remained present for the duration of the WPVTs. Participants were instructed to use either their index or middle finger on their preferred hand to respond and to rest their finger on the ‘down’ arrow in between trials. They were also instructed to respond as quickly and accurately as possible. On the rWPVT, participants were told that they would see a picture and a word at the same time. All other procedures remained the same as described for the aWPVT. Participants first completed eight practice trials (two Targets, two Semantic foils, two Phonological foils, and two Unrelated foils) for each WPVT version. Visual and verbal feedback was provided during WPVT practice trials only. No feedback was provided during the experimental WPVT trials.

### 2.3 Statistical analysis

Kendall’s Tau (one-tailed) was used to assess the relationship between established receptive language tasks and WPVT performance due to small sample sizes, violations of normality, and potential outliers. Kendall’s tau has been observed to approach the normal distribution more quickly compared to Spearman’s rho, which is especially useful with small sample sizes (53)^2^. Associations were assessed among multiple pairs of assessments: overall aWPVT accuracy and *CAT* auditory comprehension standard scores, overall rWPVT accuracy was and *CAT* reading comprehension standard scores, WPVT a-prime semantic trial statistics and *PPT* proportion accuracy, rWPVT phonological trial a-prime statistics and composite a-prime statistic for the *PALPA*s (i.e., average of a-prime statistics calculated for the two subtests), and aWPVT phonological trial a-prime statistics and *SAPA2* a-prime statistic.

Non-parametric Wilcoxon signed rank tests were employed to investigate accuracy performance between WPVTs and the *BNT*. Small sample sizes, violations of normality, and potential outliers resulted in selection of this nonparametric within-group analysis. Previous research has shown no difference in semantic errors made during picture naming and on the WPVT (9), but there has not yet been investigation of the same link for phonological errors. Number of errors on semantic and phonological trials on WPVTs were compared with number of semantic and phonological [i.e., phonologically related nonword and formal errors combined, as defined by the Philadelphia Naming Test scoring conventions (14, 48)] errors produced on the *BNT*, respectively. Inclusion of both formal and phonological nonword paraphasias was justified based on claims that formal paraphasic production reflects processing at the lexical level while phonological nonword paraphasic production reflects a breakdown in the connections between lexical and phonological encoding (35)(54).

Linear mixed effect and logistic mixed effect models were used to investigate how established measures of language processing predicted WPVT accuracy and response latency using packages *lme4* (55) and *lmerTest* (56) in rStudio (R v. 3.6.3).

## 3 Results

Twenty-seven IWAs were screened, and 16 met inclusion criteria and completed all study related tasks. Two IWAs identified as African American, and the remaining participants identified as Caucasian. See Table 1 for participant demographic information, Table 2 for performance on receptive and expressive semantic and phonological tasks, and Tables 3, 4 for WPVT accuracy and response latency performance.

**Table 1:** Participant demographic information.

Subject	Sex	Age	Education (Years)	Months Post Stroke	CAT	CAT AC	CAT RC	MSS
100	M	73	13	96	47.63	45	51	3.50
101	M	69	14	99	55.13	50	59	0.50
102	M	80	18	214	50.63	48	41	0
103	M	39	17	66	50.50	47	50	0.50
104	F	49	12.50	7	58.88	74	62	0.50
105	M	70	16	47	46	39	45	1.50
106	M	72	13	34	47	42	48	2.00
107	F	53	14	25	60.88	59	55	0
108	M	55	17	6	46.38	46	44	1.50
109	F	73	16	42	61	60	65	0
110	M	56	19	275	56.38	51	53	1.00
111	F	78	12	87	56	49	53	1.50
112	M	64	16	64	58.50	54	54	1.50
114	M	66	17	190	57	59	51	0.50
115	F	72	16	6	62.75	59	53	0
116	M	87	16	20	61.25	59	58	0
*Summary*	5 *F*, 11 *M*	*M = 66 ± 12.62*	*M = *15.41* ± * 2.08	*M = *79.88* ± *80.31	*M = *54.74* ±* 5.86	*M = *52.56* ± *8.76	*M = *52.63* ±* 6.43	*Median = *0.50

M, Male; F, Female; CAT, Comprehensive Aphasia Test; CAT AC, Comprehensive Aphasia Test Auditory Comprehension Score; CAT RC, Comprehensive Aphasia Test Reading Comprehension Score; MSS, Motor Speech Severity Rating (0–7); M, Mean.

**Table 2:** Raw scores on receptive and expressive language measures.

Subject	Receptive Language Measures				Expressive Language Measures					
	PPT (out of 52)	SAPA2 (out of 59)	PALPA 15 (out of 57)	PALPA 25 (out of 120)	BNT (out of 60)	s-weight	*p*-weight	Semantic Paraphasias	Formal Paraphasias	Phonological Nonword Paraphasias
100	50	35	32	106	18	0.0282	0.0082	1	8	24
101	50	50	45	113	43	0.0375	0.0138	2	0	12
102	43	40	33	89	33	0.0176	0.0250	4	1	2
103	47	46	35	109	39	0.0300	0.0163	1	2	9
104	52	57	54	119	50	0.0400	0.0188	0	1	5
105	42	32	33	108	3	0.0004	0.0204	4	7	3
106	50	42	40	114	17	0.0188	0.0101	1	5	20
107	52	52	49	119	54	0.0400	0.0188	0	0	6
108	48	53	35	76	14	0.0113	0.0213	7	3	3
109	51	55	52	119	45	0.0257	0.0344	1	0	0
110	48	50	35	118	41	0.0394	0.0144	2	0	7
111	49	43	39	116	41	0.0319	0.0194	0	3	4
112	48	57	56	120	50	0.0400	0.0163	0	2	8
114	51	46	38	102	43	0.0269	0.0282	2	2	0
115	51	51	44	119	58	0.0375	0.0375	0	0	0
116	49	54	53	118	48	0.0269	0.0282	2	0	1
*M ± SD*	48.81 ± 2.88	47.69 ± 7.58	42.06 ± 8.41	110.31 ± 12.37	37.31 ± 15.96	0.03 ± 0.01	0.02 ± 0.01	1.69 ± 1.92	2.13 ± 2.55	6.5 ± 7.01

PPT, Pyramids and Palm Trees; SAPA2, Standardized Assessment of Phonology in Aphasia, Subtest 2; PALPA, Psycholinguistic Assessment of Language Processing in Aphasia; BNT, Boston Naming Test; M, Mean; SD, Standard Deviation.

**Table 3:** aWPVT raw accuracy and mean response latency performance.

Subject	Accuracy (out of 60)			Reaction Time (ms)		
	Overall	Semantic	Phonological	Overall	Semantic	Phonological
100	33	51	44	1867.78	1965.73	2005.02
101	51	55	57	1207.24	1231.84	1272.93
102	30	43	54	3751.78	4447.63	3716.50
103	50	53	58	1336.70	1529.74	1316.28
104	57	58	60	1984.02	2068.07	1901.45
105	29	41	51	1761.81	1842.54	1802.75
106	42	48	54	1570.61	1720.73	1614.44
107	57	59	60	1022.19	1020.78	1073.22
108	42	49	60	1644.67	1922.53	1508.50
109	53	55	60	1306.89	1364.71	1370.97
110	52	54	59	1359.34	1578.48	1378.15
111	54	59	56	1645.42	1524.61	1859
112	59	59	60	1246.56	1234.59	1315.48
114	51	55	55	1465.52	1500.16	1563.47
115	56	59	59	1369.11	1316.08	1455.24
116	44	47	58	1890.33	2041.15	2027.09
*M * *±* * SD*	47.50 ± 9.80	52.81 ± 5.81	56.56 ± 4.32	1651.87 ± 622.86	1769.34 ± 780.46	1698.78 ± 608.13

M, Mean; SD, Standard Deviation.

**Table 4:** rWPVT raw accuracy and mean response latency performance.

Subject	Accuracy (out of 60)			Reaction Time (ms)		
	Overall	Semantic	Phonological	Overall	Semantic	Phonological
100	40	57	52	2092.98	2227.91	2093.60
101	50	54	56	1024.76	1106.57	1059.57
102	25	45	40	3305.59	3578.58	3218.40
103	51	54	57	1322.55	1396.70	1260.68
104	50	57	60	1476.12	1459.54	1502.18
105	15	22	44	2399.71	3024.77	2340.48
106	47	49	57	2139.34	2549.04	2286.42
107	57	59	60	1226.66	1258.15	1160.63
108	27	47	54	5471.42	6213.80	5780.37
109	56	56	60	1368.19	1443.50	1335.35
110	48	50	57	1499.77	1868.76	1411.72
111	56	58	59	1404.17	1443.90	1334.17
112	57	58	60	1186.09	1224.60	1186.37
114	44	51	56	1550.37	1479.02	1617.18
115	52	53	60	1480.97	1649.87	1330.72
116	42	42	59	2093.75	2645.52	2012.34
*M * *±* * SD*	44.81 ± 12.48	50.75 ± 9.16	55.69 ± 5.88	1940.15 ± 1105.01	2160.64 ± 1298.50	1933.14 ± 1177.06

M, Mean; SD, Standard Deviation.

### 3.1 WPVT and aphasia severity

Aphasia severity (as measured by the CAT mean modality *T* score) was not significantly correlated with overall WPVT response latency (aWPVT: *τb *= −0.18, *p* = 0.35; rWPVT: *τb* = −0.3, *p* = 0.12) nor latencies on target (aWPVT: *τb* = −0.23, *p* = 0.23; rWPVT: *τb* = −0.3, *p* = 0.12), semantic (aWPVT: *τb* = −0.28, *p* = 0.14; rWPVT: *τb* = −0.28, *p* = 0.14), or phonological (aWPVT: *τb* = −0.12, *p* = 0.56; rWPVT: *τb* = −0.35, *p* = 0.06) trials. However, aWPVT overall accuracy (aWPVT: *τb* = 0.53, *p* = 0.004; rWPVT: *τb* = 0.43, *p* = 0.02) as well as accuracy on target (aWPVT: *τb* = 0.48, *p* = 0.01; rWPVT: *τb* = 0.45, *p* = 0.015), aWPVT semantic (aWPVT: *τb* = 0.54, *p* = 0.003; rWPVT: *τb* = 0.40, *p* = 0.03), and phonological (aWPVT: *τb* = 0.47, *p* = 0.01; rWPVT: *τb* = 0.49, *p* = 0.01) trials were significantly correlated with aphasia severity (corrected *ɑ* = 0.017). Participants with less severe aphasia tended to be more accurate on WPVTs.

### 3.2 WPVT and receptive language

WPVT overall accuracy was significantly positively correlated with overall comprehension ability as measured by the *CAT* (aWPVT: *τb* = 0.58, *p* = 0.001; rWPVT: *τb* = 0.51, *p* = 0.004). Accuracy on both aWPVT and rWPVT semantic trials was significantly positively correlated with semantic abilities as measured by *PPT* (corrected *α* = 0.025; aWPVT: *τb* = 0.42, *p* = 0.014; rWPVT: *τb* = 0.43, *p* = 0.013). aWPVT and rWPVT phonological trials were significantly positively correlated with phonological abilities as measured by the *SAPA2* (auditory phonology; *τb* = 0.61, *p* = 0.001) and combined *PALPA* (reading phonology; *τb* = 0.60, *p* = 0.001). Across all correlations, it was demonstrated that IWAs who had better language input processing tended to score higher on the WPVT.

### 3.3 WPVT and expressive language

Inter- and intra-rater reliability were calculated on 25% of samples (i.e., 4 participants) to assess accuracy of *BNT* overall score as well as the number of semantic, formal, and phonologically related nonword paraphasias using absolute agreement intraclass correlations (mixed two-way consistency) (57). A blinded certified speech-language pathologist was trained in the *BNT* scoring schema and served as the second rater. Reliability was observed to be excellent for overall *BNT* score (Inter-rater: ICC = 0.997, 95% CI [0.96, 1.00]; Intra-rater: ICC = 0.99, 95% CI [0.93, 1.00]), number of formal paraphasias (Inter-rater: ICC = 0.98, 95% CI [.76, 1.00]; Intra-rater: ICC = 0.91, 95% CI [0.03, 0.99]), and number of phonologically related nonword paraphasias (Inter-rater: ICC = 0.95, 95% CI [0.56, 1.00]; Intra-rater: ICC = 0.997, 95% CI [0.95, 1.00]). Agreement was moderate for number of semantic paraphasias (Inter-rater: ICC = 0.56, 95% CI [−0.20, 0.96]; Intra-rater: ICC = 0.68, 95% CI [−0.75, 0.98]).

There was a significant difference in semantic errors made on the *BNT* and both WPVTs, with more semantic errors made on the verification task compared to the confrontation naming task (corrected *α* = 0.025; aWPVT: *z* = −3.53, *p* < 0.001; rWPVT: *z* = −3.52, *p* < 0.001). Even when mixed paraphasias are included with semantic paraphasias, more semantic errors are still observed during verification than during naming (aWPVT: *z* = −2.89, *p* = 0.004; rWPVT: *z* = −3.22, *p *= 0.001). There was also a significant difference between phonological paraphasias and aWPVT phonological trial errors (corrected *α* = 0.025; *z* = 2.62, *p* = 0.009). More phonological paraphasias were produced during confrontation naming compared to verification. No significant difference was observed in phonological errors made on the rWPVT and the *BNT* (*z* = 1.85, *p* = 0.064).

### 3.4 Predicting WPVT performance

Since sample size was small, only semantic (s-weight and *PPT* scores) and phonological tasks (*SAPA2* [for aWPVT], and *PALPA* [for rWPVT]) were entered as predictors into models of item-level data to avoid overfitting. Receptive and expressive language variables were standardized, and response latencies were log-transformed. Participants were included as random intercepts across all models since item-level performance was nested within participants.

**Figure 4:**
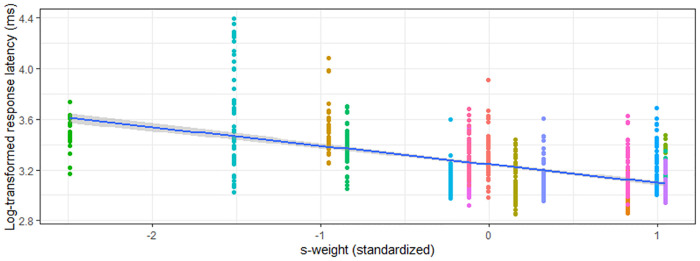
Relationship between s-weight (standardized) and rWPVT semantic trial response latency (log-transformed) across participants (represented by different colors).

Models predicting verification semantic accuracy were significant for both the aWPVT [*Χ*^2^(2) = 20.19, *p* < 0.001] and rWPVT [*Χ*^2^(2) = 20.88, *p* < 0.001]. S-weight emerged as a significant predictor in both models, and PPT score was a significant predictor for rWPVT semantic trial accuracy (Table 5). Semantic latency models were significant for rWPVT [*Χ*^2^(2) = 18.25, *p* < 0.001] and the aWPVT [*Χ*^2^(2) = 6.53, *p* = 0.04]. S-weight was the only significant predictor in the rWPVT model (Figure 4).

**Table 5:** Summary of WPVT semantic trial logistic mixed effect models.

Factor	Estimate	SE	Z-value	*p*-value	CI (95%)
aWPVT					
Intercept	2.28	0.14	16.57	<0.001	
s-weight*	0.55	0.16	3.40	<0.001	0.23, 0.87
PPT	0.21	0.15	1.39	0.16	−0.08, 0.50
rWPVT					
Intercept	2.05	0.13	15.41	<0.001	
s-weight*	0.57	0.16	3.53	<0.001	0.25, 0.89
PPT*	0.34	0.16	2.19	0.03	0.04, 0.65

*indicates a significant effect (*p* < 0.05).

**Figure 5:**
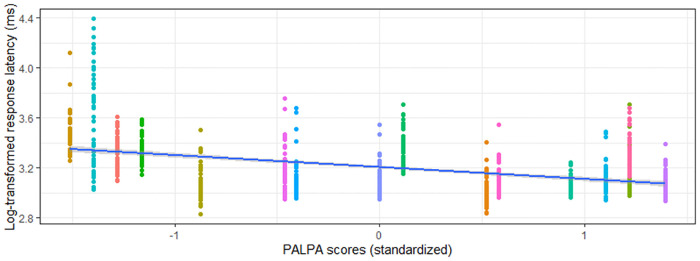
Relationship between PALPA (standardized) and rWPVT phonological trial response latency (log-transformed) across participants (represented by different colors).

**Figure 6:**
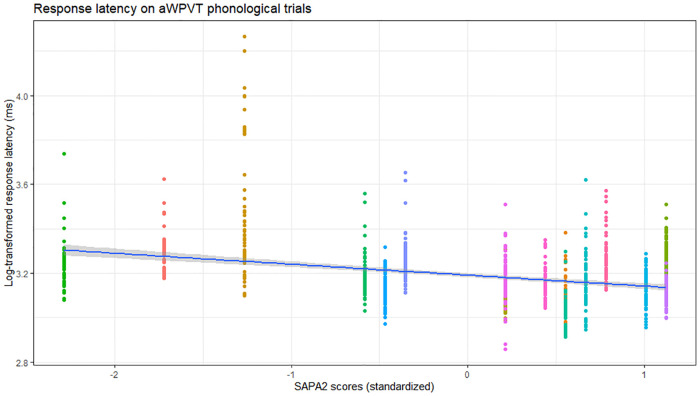
Relationship between SAPA2 (standardized) and aWPVT phonological trial response latency (log-transformed) across participants (represented by different colors).

**Figure 7:**
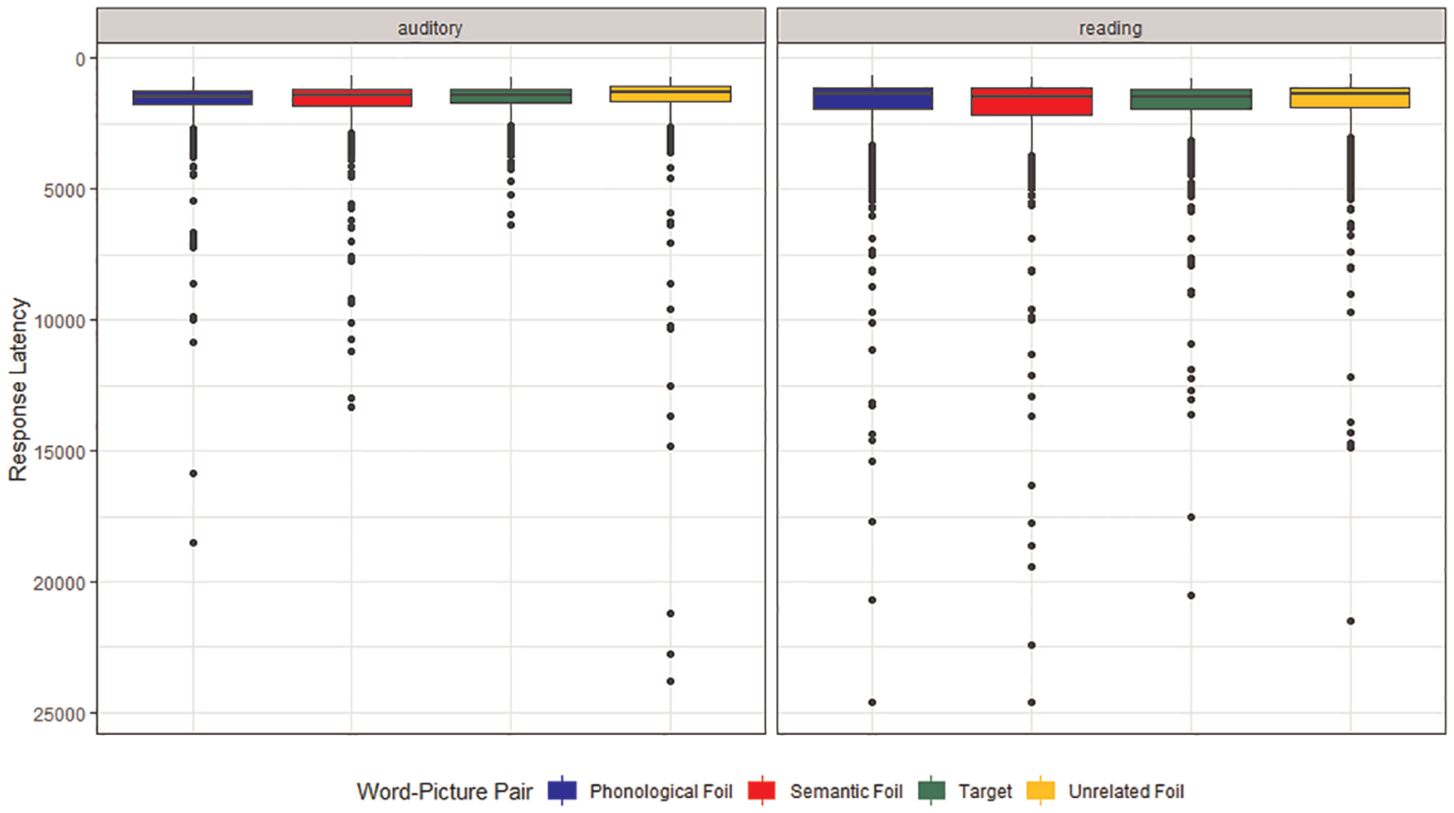
Response latency on auditory and reading verification tasks by word-picture pair.

Phonological verification accuracy models were significant for both aWPVT [*Χ*^2^(2) = 12.48, *p* = 0.002] and rWPVT [*Χ*^2^(2) = 13.31, *p* = 0.001]. For both models, the receptive language measures (aWPVT = *SAPA2*, rWPVT = *PALPA*) were significant predictors (Table 6). rWPVT phonological response latency model was significant [*Χ*^2^(2) = 8.29, *p* = 0.02] along with the aWPVT model [*Χ*^2^(2) = 5.95, *p* = 0.05]. Again, *PALPA* and *SAPA2* scores emerged as significant predictors of WPVT phonological trial reaction times (Tables 7, 8 & Figures 5–7).

**Table 6:** Summary of WPVT phonological trial logistic mixed effect models.

Factor	Estimate	SE	Z-value	*p*-value	CI (95%)
aWPVT					
Intercept	3.27	0.29	11.22	<0.001	
*p*-weight	0.03	0.26	0.12	0.90	−0.48, 0.54
SAPA2*	0.92	0.26	3.50	<0.001	0.41, 1.44
rWPVT					
Intercept	3.20	0.32	10.02	<0.001	
*p*-weight	−0.04	0.30	−0.12	0.91	−0.62, 0.55
PALPA*	1.15	0.30	3.83	<0.001	0.56, 1.73

*indicates a significant effect (*p* < 0.05).

**Table 7:** Summary of rWPVT response latency linear mixed effect models.

Factor	Estimate	SE	df	*t*-value	*p*-value	CI (95%)
Semantic trials (conditional R^2^ = 0.47, marginal R^2^ = 0.28)						
Intercept	3.24	0.03	12.86	126.44	<0.001	
s-weight*	−0.13	0.03	12.94	−3.72	0.003	−0.19, −0.06
PPT	−0.01	0.03	12.93	−0.39	0.70	−0.08, 0.05
Phonological trials (conditional R^2^ = 0.55, marginal R^2^ = 0.19)						
Intercept	3.21	0.03	13.01	92.97	<0.001	
*p*-weight	0.03	0.04	12.99	0.71	0.49	−0.04, 0.09
PALPA*	−0.11	0.04	13.04	−2.97	0.01	−0.18, −0.04

*indicates a significant effect (*p* < 0.05).

**Table 8:** Summary of aWPVT response latency linear mixed effect models.

Factor	Estimate	SE	df	*t*-value	*p*-value	CI (95%)
Semantic trials (conditional R^2^ = 0.47, marginal R^2^ = 0.13)						
Intercept	3.20	0.03	12.96	119.17	<0.001	
s-weight	−0.02	0.04	12.98	−0.67	0.51	−0.09, 0.05
PPT	−0.05	0.04	13.01	−1.41	0.18	−0.12, 0.02
Phonological trials (conditional R^2^ = 0.45, marginal R^2^ = 0.12)						
Intercept	3.19	0.02	13.01	142.02	<0.001	
*p*-weight	0.02	0.02	13.00	1.03	0.32	−0.02, 0.07
SAPA2*	−0.06	0.02	13.04	−2.41	0.03	−0.10, −0.01

*indicates a significant effect (*p* < 0.05).

## 4 Discussion

The current study aimed to elucidate the relationship among the WPVT and receptive and expressive language, namely semantic and phonological processing, at the single word level. Both WPVTs were found to be significantly positively correlated with other established measures of receptive language, including broad measures of language comprehension (auditory and reading) and semantic and phonological processing, aligning with previous work reporting the paradigm as a measure of receptive language (1, 4–6, 9, 16). That is, IWAs with better receptive language skills tended to score higher on the WPVT than those with poorer receptive language skills. Breese and Hillis (4) observed that their WPVT identified more IWAs as demonstrating impaired auditory comprehension compared to a multiple-choice picture matching task. To the best of our knowledge, the current study is the first to compare performance on a reading verification task with an established measure of reading comprehension, observing a significant positive correlation between measures. The moderate correlations between WPVTs and *CAT* comprehension subtests is expected since WPVTs assessed receptive language at the single word level whereas *CAT* subtests comprise single word, sentence, and discourse (for auditory comprehension) levels.

### 4.1 Verification and semantic processing

WPVT semantic trial accuracy was significantly positively correlated with measures of conceptual semantic processing, and models predicting semantic verification response accuracy and latency were significant. In these predictive models, s-weight, which is a measure of the connections between the conceptual and lexical stages of word retrieval according to Dell’s two-stage model (14), was the only significant predictor for auditory verification. For reading verification, both s-weight and PPT scores were significant predictors. Previous research supported the WPVT in targeting semantic and lexical-semantic systems (2, 5, 9, 16, 58, 59), including both receptive *and* expressive semantic processes. Stadthagen-Gonzalez and colleagues (2) concluded that WPVTs assess pre-lexical semantic processing in the context of naming as evidenced by significant correlations between WPVT performance with naming stimuli’s perceptual and conceptual characteristics and a lack of significant correlations with lexical characteristics. However, the current study finds significant correlations with other receptive language tasks, and it is posited that the WPVTs utilized in the current study may have provided a more linguistically-rich context by the inclusion of semantic and phonological foils compared to the task utilized by Stadthagen-Gonzalez and colleagues (2) in which only unrelated foils were included. Verification appears to assess phonological decoding onto the lexicon and semantic encoding/decoding from the lexicon based on the verification tasks’ association with measures of semantic (i.e., s-weight, PPT accuracy) and phonological (i.e., SAPA2, PALPA) processing.

### 4.2 Verification and phonological processing

The current study also uniquely assessed the relationship between WPVTs and receptive phonological processing. A significant positive correlation was observed between WPVT phonological accuracy and both reading and auditory phonological processing tasks, which was assessed *via* lexical decision, rhyme judgment, and minimal pairs (auditory only). Performance on phonological and orthographic decoding tasks were significant predictors of phonological verification response accuracy and latency. Previous WPVTs have included auditory phonological foils (1, 9, 16), but these studies focused primarily on semantic rather than phonological performance. Rogalsky and colleagues (16) reported that WPVTs are more sensitive to semantic over phonological deficits as evidenced by greater accuracy across phonological word-picture pairs compared to semantic pairs in a group of individuals with acute post-stroke aphasia. The researchers concluded that the superior phonological performance was a result of a more bilaterally supported phonological system (60–62) and more left-lateralized semantic system. Despite the claim that WPVTs may be better suited for detecting semantic impairments over phonological ones, the current study provides evidence that the paradigm can still provide information on receptive phonological processing skills.

### 4.3 Verification versus naming: recognition versus recall

Comparing WPVT errors and paraphasia production on the BNT, a confrontation naming task designed to assess lexical retrieval, findings did not align with our hypotheses. More semantic errors were made during verification than during naming, whereas previous research reported no difference in errors between these two tasks (8, 9). Discrepant findings support differences in processes theorized to be engaged during each task rather than due to methodological differences. In terms of methodology, Zezinka and colleagues (8) based their definition of semantic paraphasia on that reported by Hillis and colleagues (9). To better operationalize the definition of semantic paraphasia in the current study, classification of five paraphasia subtypes were adopted from the *Philadelphia Naming Test* (48) and Dell and colleagues (14, 34). One difference between the two definitions used is the classification of mixed paraphasias. Hillis (9) and Zezinka (8) and their respective colleagues included mixed paraphasias in their definition of semantic paraphasias, whereas mixed paraphasias are classified separately from semantic in the *Philadelphia Naming Test*. However, even when mixed paraphasias were included with semantic paraphasias in the current study, more semantic errors were still observed during verification than during naming (aWPVT: *z* = −2.89, *p* = 0.004; rWPVT: *z* = −3.22, *p *= 0.001).

Rather, differences in semantic errors between these two tasks are likely driven by the demand of distinct cognitive-linguistic processes on semantic processing. Verification requires recognition while confrontation naming relies more heavily on recall. Lexical retrieval may occur implicitly or covertly during verification, but this claim is only speculative based on the current study design. Use of functional neuroimaging during testing or discussion of strategies used by participants at study conclusion may further elucidate this claim. Under the notion that the language system is interactive (14), both top-down and bottom-up processes influence language performance; however, one may argue that recall and recognition engage these processes to a different degree. Word recall (i.e., picture naming) likely relies more on top-down conceptuo-linguistic processing while recognition (i.e., WPVT) likely utilizes more bottom-up perceptuo-linguistic processes. Findings from Mirman and colleagues (63, 64) provide further support for distinct *processes*, not distinct *semantics*, with recall and recognition as semantic recognition and recall loaded on separate factors. They also observed different brain activation patterns for semantic processing during naming (left anterior temporal lobe) and recognition (white matter tracts connecting frontal lobe to other cortical regions). Thus, the difference in WPVT and naming semantic performance is not entirely at odds with models of modality-independent semantic organization. According the *O.U.C.H* (15)., damage to the hub would result in multi-modal semantic deficits, such as in word-picture matching and naming. However, damage may also be sustained to the processes that access the hub, such as *via* lexical or visual representations. Thus, if damage were sustained to one of these processes, semantic performance deficits would appear modality specific.

Different relationships were observed between phonological paraphasic errors and WPVT phonological errors depending on verification modality. Significantly more phonological paraphasias were produced on the *BNT* compared to errors made on phonological trials of the aWPVT. According to Martin and Saffran’s Model 1 (35), a single representation of semantics, lexicons, and phonology are shared for language comprehension and production. In their coupled model (Model 2), separate input and output phonological codes are connected while lexical-semantic representations are shared, which appears to be a shared feature among other language processing models (13, 37). In this coupled model, activation can spread from input to output phonological codes, and vice versa, but the degree of activation flow in each direction is task specific. For instance, activation will be weaker for input phonological codes compared to output codes during word retrieval since word retrieval is a language production-motivated task rather than a language comprehension-motivated task. Additionally, if (expressive) phonological representations are damaged, receptive performance should be relatively preserved due to interactive activation “stabilization” provided by lexical and semantic representations for the impaired phonological codes (35). Thus, more phonological errors would be expected during production compared to auditory comprehension, which was the case in the current study. Additionally, the more bilateral hemispheric activation according to the dual stream model (60–62) may indicate that auditory comprehension is more preserved compared to lexical retrieval, which appears to be more left-lateralized (65).

In contrast with auditory verification findings, phonological errors made during reading verification and during naming did not differ. In a coupled phonological network according to Indefrey and Levelt (13), relatively equal degrees of damage to the orthographic input and encoding output phonological processes would need to occur to mimic study findings. This pattern would result in relatively intact auditory phonological processing compared to phonological encoding, which was observed in the current study as well. In their model, receptive orthographic codes can be converted to receptive phonological codes, which share a connection to expressive phonemes, or processed into lexical codes [e.g., Figure 2 in (13)]. Connections between receptive and expressive phonological codes are unidirectional, with information flowing from input to output. Working memory appears to be taxed more during reading than listening, as reading is a less-rehearsed skill (66, 67). Additionally, reading appears to be more left-lateralized (68, 69) and thus may be more susceptible to damage in aphasia compared to similar auditory comprehension faculties, resulting in similar error rates during naming and reading verification but more errors during naming than auditory verification. Future work incorporating cognitive tasks would help assess the relationship of cognitive processing and WPVT performance.

### 4.4 Limitations

The current study is not without limitations. First, the sample of individuals with aphasia is relatively small and homogenous in terms of demographic characteristics. A larger, more heterogenous sample is needed to replicate and validate study findings. Also, exploration into the cognitive variables that may influence verification performance should be completed to further understand the interplay between cognitive and language systems. Finally, verification task stimuli would benefit from further investigation. Recent concerns with the *BNT* stimuli have been raised, including differential age effects in healthy adults (70–72), and the stimuli is copyrighted, making it not as accessible to clinicians and researchers as compared to tests that are freely available, including the *Philadelphia Naming Test* (48). In addition to perhaps pursuing a new set of stimuli, determining the minimum number of items and administration methods, such as computer adaptive testing, to achieve optimal psychometric value should be explored.

### 4.5 Role of WPVT in improving individualized aphasia care

WPVTs provide a more sensitive measure of receptive language (compared to multiple-choice tasks) and linguistic processes that support receptive and expressive language at the word level. Specifically, accuracy and reaction time on WPVT semantic trials provides insight on pre-lexical semantic processes, including the connections between conceptual semantic information and the lexicon, which is shared between receptive and expressive language processes (13). Accuracy and response latency on WPVT phonological trials shed light on phonological *decoding* (i.e., receptive language). Thus, including a verification task with well-controlled linguistic variables in aphasia assessment can aid in impairment localization and inform individualized, targeted intervention.

Recent advances in anomia treatment research identify linguistic processes, namely semantics and phonology, as important predictors to treatment response. Kristinsson and colleagues (73) found that individuals with aphasia and more intact phonological and semantic processing responded well to semantically based naming treatments. Individuals with aphasia who responded well to phonologically based treatments tended to have more severe aphasia and apraxia of speech. Thus, more standardized assessments are needed that characterize patients’ language abilities, including the processes theorized to support gross language skills (i.e., reading and auditory comprehension, naming, writing), in order to more successfully tailor anomia treatment to individual patients with aphasia.

There is a shift in aphasia research to identify speech-language strengths and weaknesses and their severity instead of using aphasia classification profiles since all individuals classified as belonging to the same aphasia subtype may not present with the same symptoms, or they may not be classified completely (74). Standardized assessments may over-represent certain language skills over others in their composite scores (22), and many batteries and shorter assessments do not disclose sufficient information on stimuli development, including lexical variables controlled for and theoretical models referenced to inform test development. The *CAT* (24) one battery that does describe the linguistic variables controlled for and purposefully manipulated to increase difficulty across test items. Completion of the entire *CAT* language battery can take around 45 min, which is not practical for patients in acute care settings. To our knowledge, the *ScreeLing* (75, 76), which is only available in Dutch, is the only short, standardized assessment that provides information on phonology and semantics. We argue that if theoretical, empirical models are critical to understand typical and disordered language processing, then our assessments should be transparent in how they are informed by linguistic models and variables *known* to influence performance and treatment response. Further, as aphasia treatment response research advances, and more variables are identified to predict treatment response, our assessments need to be refined to capture those predictors efficiently and soundly.

Using WPVTs, information on semantic and phonological processing can be gleaned from a single task and used to inform individualized anomia treatment planning. The use of button press for responding allows easy collection of response time as well as eliminates demands on verbal output in the cases of significant motor speech impairment that may interfere with language assessment. With continued psychometric development, standardized WPVTs that collect response accuracy and latency would be a strong tool added to clinicians’ and researchers’ aphasia assessment inventory.

## Data Availability

The raw data supporting the conclusions of this article will be made available by the authors upon request.

## Footnotes

^1^Though researchers implicate interaction between orthography and phonology during visual lexical decision, the focus of the task is believed to be within the orthographic lexicon.

^2^According to Puth, Neuhäuser, and Ruxton (54), if there are many ties and large *р* in the dataset, then Spearman’s rho may provide more accurate confidence interval estimates.
